# Drug Repurposing for Duchenne Muscular Dystrophy: The Monoamine Oxidase B Inhibitor Safinamide Ameliorates the Pathological Phenotype in *mdx* Mice and in Myogenic Cultures From DMD Patients

**DOI:** 10.3389/fphys.2018.01087

**Published:** 2018-08-14

**Authors:** Libero Vitiello, Manuela Marabita, Elisa Sorato, Leonardo Nogara, Giada Forestan, Vincent Mouly, Leonardo Salviati, Manuel Acosta, Bert Blaauw, Marcella Canton

**Affiliations:** ^1^Department of Biology, University of Padova, Padova, Italy; ^2^Interuniversity Institute of Myology, Padova, Italy; ^3^Venetian Institute of Molecular Medicine (VIMM), Padova, Italy; ^4^Department of Biomedical Sciences, University of Padova, Padova, Italy; ^5^UMRS 974 UPMC-INSERM, Center for Research in Myology, Paris, France; ^6^Fondazione Istituto di Ricerca Pediatrica Città della Speranza – IRP, Padova, Italy

**Keywords:** muscular dystrophy, mitochondrial ROS, monoamine oxidase, oxidative stress, safinamide, DMD, *mdx*

## Abstract

Oxidative stress and mitochondrial dysfunction play a crucial role in the pathophysiology of muscular dystrophies. We previously reported that the mitochondrial enzyme monoamine oxidase (MAO) is a relevant source of reactive oxygen species (ROS) not only in murine models of muscular dystrophy, in which it directly contributes to contractile impairment, but also in muscle cells from collagen VI-deficient patients. Here, we now assessed the efficacy of a novel MAO-B inhibitor, safinamide, using *in vivo* and *in vitro* models of Duchenne muscular dystrophy (DMD). Specifically, we found that administration of safinamide in 3-month-old *mdx* mice reduced myofiber damage and oxidative stress and improved muscle functionality. *In vitro* studies with myogenic cultures from *mdx* mice and DMD patients showed that even cultured dystrophic myoblasts were more susceptible to oxidative stress than matching cells from healthy donors. Indeed, upon exposure to the MAO substrate tyramine or to hydrogen peroxide, DMD muscle cells displayed a rise in ROS levels and a consequent mitochondrial depolarization. Remarkably, both phenotypes normalized when cultures were treated with safinamide. Given that safinamide is already in clinical use for neurological disorders, our findings could pave the way toward a promising translation into clinical trials for DMD patients as a classic case of drug repurposing.

## Introduction

Duchenne muscular dystrophy (DMD) is the one of the most common and severe forms of inherited muscular dystrophies. Despite improvements in palliative and support care, it is still invariably lethal because of cardiac-respiratory failure (Davies and Nowak, 2006; Shin et al., 2013), usually between the second and third decade of life. The only clinically available therapies rely on steroid anti-inflammatory molecules, although a number of other drugs are presently in clinical trials (Rosenberg et al., 2015; Guiraud and Davies, 2017). Two genetic-based drugs have recently been approved for clinical use (Fairclough et al., 2013), but they are aimed at patients bearing defined mutations and their efficacy is still marginal.

Even though the genetic bases of most inherited muscular dystrophies have been characterized, the underlying pathogenic mechanisms remain somewhat elusive. Mitochondrial dysfunction has been shown to play a key role (Irwin et al., 2003; Angelin et al., 2007; Merlini et al., 2008; Millay et al., 2008; Palma et al., 2009; Menazza et al., 2010; Sorato et al., 2014; Chartier et al., 2015), although in the case of DMD, the mechanisms connecting lack of dystrophin, a sub-sarcolemmal protein, to mitochondrial alterations are unclear. A likely candidate is oxidative stress, as highlighted by several studies (Disatnik et al., 1998; Rando, 2002; Tidball and Wehling-Henricks, 2007; Williams and Allen, 2007; Whitehead et al., 2008; Lawler, 2011; Canton et al., 2014). In this regard, we previously demonstrated that monoamine oxidase (MAO), a mitochondrial enzyme widely studied in the central nervous system, is an essential source of reactive oxygen species (ROS) in dystrophic muscles (Menazza et al., 2010). The two isoforms of MAO, A and B, located in the outer mitochondrial membrane, catalyze the oxidative deamination of biogenic amines generating aldehydes, ammonia, and H_2_O_2_, which are removed by enzymatic scavengers in physiological conditions (Youdim et al., 2006). We have already reported that MAO expression and activity increase in muscles from two murine models of muscular dystrophies, *mdx* for DMD and *Col6a1*^-/-^ mice for collagen VI-related myopathies (Menazza et al., 2010). This results in excessive levels of H_2_O_2_, which in turn alters the redox homeostasis and causes myofibrillar protein oxidation, hampering contractile function. Importantly, treatment with pargyline, an inhibitor of both MAO isoforms, reduced oxidation of tropomyosin and led to improvements in the phenotype of dystrophic mice (Menazza et al., 2010). The involvement of MAO in muscular dystrophy has also been seen in *in vitro* myoblasts cultures obtained from patients with collagen VI myopathies (Sorato et al., 2014). Specifically, in these cells, pargyline treatment reduced ROS accumulation and mitochondrial dysfunction, while normalizing the occurrence of apoptosis. These findings proved that MAO-dependent ROS accumulation is directly linked to mitochondrial dysfunction and suggested that it is upstream of the opening of the permeability transition pore (Sorato et al., 2014).

In our previous researches, pargyline was chosen as a “proof-of-principle” molecule in assessing MAO role in muscular dystrophy, thanks to its strong and irreversible inhibitory effect. However, its use in patients is hampered by significant side effects and its clinical use has been discontinued in favor of different, well-tolerated MAO inhibitors that are now commonly used in clinics for neurological disorders (Youdim et al., 2006). Among these, inhibitors specific for MAO-B, which are mainly used for treatment of Parkinson disease, have the advantage of not causing the severe side effects seen with drugs inhibiting MAO-A. In the present study, we investigate for the first time the specific role of MAO-B in cultured muscle cells from DMD patients and in skeletal muscles of *mdx* mice, by using the novel pharmacological MAO-B inhibitor safinamide. Our data demonstrate that accumulation of ROS related to MAO-B activity not only plays a crucial role in the loss of cell viability and contractile impairment of dystrophic skeletal muscles but also in the mitochondrial dysfunction occurring in DMD myogenic cultures, thereby pointing at safinamide as a promising candidate for DMD therapy.

## Materials and Methods

### Chemicals

Safinamide was kindly provided by Zambon SpA (batch 14A03C0483) and dissolved in water or phosphate-buffered saline (PBS). Unless otherwise stated, all chemicals used were purchased from Sigma-Aldrich.

### Mice and Safinamide *in vivo* Treatments

Wild-type C57BL/10ScSnJ and *mdx* mice (C57BL/10ScSn-*Dmd^mdx^*/J) were obtained from Charles River and Jackson Laboratories, respectively, and were bred and maintained in the animal facility of the Venetian Institute for Molecular Medicine (VIMM). Safinamide (20 or 40 mg/kg/day), prednisolone (2 mg/kg/day), or vehicle (PBS) were administered by daily intra peritoneal injection for 7 or 30 days in 3-month-old *mdx* and C57BL/10ScSn male mice. The doses of safinamide were chosen based on preliminary data obtained by ZambonGroup. At the end of the treatment, animals were first analyzed for force measurements and then sacrificed for muscle harvesting. Collected samples were then stored in liquid nitrogen until use. All *in vivo* experiments were approved by the Institutional Animal Care and Use Committee of the University of Padova.

### Muscle Functional Assessment

Muscle function *in vivo* was assessed for the *gastrocnemius* muscle, as described previously (Blaauw et al., 2008). Briefly, mice were anesthetized, and electrodes were placed on either side of the sciatic nerve, while the common peroneal nerve was cut. Muscle torque production was measured using a lever system (Aurora Scientific 305B). A lever arm of 2.1 mm was used for all groups, as no major differences in body weight between various groups was observed. Eccentric contractions were performed by moving the foot backward at a velocity of 40 mm/s while the gastrocnemius was stimulated with a frequency sufficient to induce full tetanic fusion (100 Hz). Contractions were repeated once every 20 s to avoid inducing fatigue.

### Analyses of Oxidation State in Skeletal Muscles

#### Dihydroethidium Staining

Dihydroethidium (DHE) is oxidized by ROS, forming ethidium bromide, which emits red fluorescence when intercalates with DNA (Benov et al., 1998). Gastrocnemius cryosections (10 μm thick) were incubated with 5 μM DHE (Sigma) for 30 min at 37°C in degassed PBS, washed twice in PBS, mounted and visualized using an inverted microscope Leica DMI6000B, as previously described (Menazza et al., 2010). Data were acquired and analyzed using Metamorph software (Universal Imaging).

#### Tropomyosin Oxidation

Assessment of tropomyosin oxidation was carried out by western blot analyses. Protein extracts were prepared homogenizing tissue samples in ice-cold PBS, pH 7.2 containing a protease inhibitor mix, subjected to PAGE, and then transferred onto nitrocellulose, as detailed in (Canton et al., 2006). Immunoblotting were then stained with an anti-tropomyosin antibody (anti-Tm, CH1 clone, Sigma-Aldrich). In anti-Tm immunoblots, the presence of high-molecular-mass bands was attributed to the oxidation-driven formation of disulphide cross-bridges (DCB), by comparing electrophoreses carried out in the absence or in the presence of β-mercaptoethanol, as previously described (Canton et al., 2006). Quantitation of Tm oxidation was performed by densitometric analysis of the bands obtained under non-reducing conditions, using the ImageJ software^1^. DCB density was normalized to the actin density in Red Ponceau, to account for differences in sample loading. Data were expressed as percentage of DCB relative to vehicle-treated *mdx* mice.

### Pathological Markers

Cross-sections (7 μm thick) were prepared and processed for hematoxylin and eosin (H&E) staining. For the morphometric analysis of myofiber CSAs, we counted between 5000 and 6000 *gastrocnemius* fibers per mouse, by means of ImageJ software. Images were acquired from least three non-sequential sections from each muscle. The same images were also analyzed to assess the percentage of centrally nucleated fibers.

Membrane permeability of skeletal fibers, used as a marker of necrosis, was visualized by immunohistochemical staining with IgG (Blaauw et al., 2008). Cryosections (10 μm thick) were incubated with anti-mouse fluorescein isothiocyanate-conjugated IgG, washed twice with PBS, mounted, and visualized with an Olympus IMT-2 inverted microscope as previously described (Menazza et al., 2010) using excitation/emission cubes of 488/525 + 25 nm bandpass.

### Creatine Kinase Assay

Serum creatine kinase (CK) was performed using a dedicated assay kit (BioAssay Systems, Hayward, CA, United States), following manufacturer’s instructions. Blood was collected by cardiac puncture from animals anesthetized with pentobarbital. Immediately after bleeding, animals were killed by cervical dislocation. Sera were separated by centrifugation at 300 ×*g* for 5 min and were stored at -80°C until use.

### Myogenic Cell Cultures

Murine myoblasts were isolated from the diaphragm of 2- to 3-month-old *mdx* mice with a standard enzymatic (Collagenase I) digestion/serial pre-plating technique.

Primary DMD and control myoblasts were kindly provided by the “Telethon Bio-Bank” (“Besta” Neurology Institute, Milan, Italy) and by the Neuroscience Department of the University of Padova. Primary myoblasts (both murine and human) were expanded in gelatin-coated dishes with proliferation medium: F12 medium (Invitrogen), 20% fetal bovine serum (FBS, Invitrogen), 5 ng/mL FGFb (Immunotools), penicillin–streptomycin mix (Invitrogen). Myoblast differentiation in myotubes was induced by switching confluent cultures to differentiate medium: high-glucose DMEM, 2% horse serum (Invitrogen), 10 mg/mL insulin (from bovine pancreas, Sigma-Aldrich), penicillin–streptomycin mix (Invitrogen). Immortalized human myoblasts, kindly provided by the Institut de Myologie (Pitié-Salpétrière Hospital, Paris, France), had been obtained as described in Mamchaoui et al. (2011) by double transduction with hTERT and cdk4. Cells were expanded in proliferation medium: M199-DMEM mix (1:4 ratio), 20% FBS, Fétuin 25 μg/ml (Invitrogen), hEGF 5 ng/ml (Immunotools), Insulin 5 μg/ml (Sigma-Aldrich), Dexamethasone 0.2 μg/ml (Sigma-Aldrich), 20% FBS, penicillin–streptomycin mix (Invitrogen). For differentiation, confluent cultures were exposed to a serum-free medium comprising DMEM (Invitrogen), insulin 10 μg/ml (Sigma-Aldrich), and penicillin–streptomycin mix (Invitrogen) for 5–7 days.

### ROS Detection

Reactive oxygen species were detected using the fluorescent probe MitoTracker Red CM-H2XRos (MTR, Molecular Probes, Eugene, OR, United States). Myoblasts were seeded onto 24-mm diameter gelatine-coated glass coverslips placed in six-well plates, and then kept for 2 days in the appropriate growth medium. For myotubes, upon reaching confluence cells were switched to the appropriate differentiation medium and kept in culture for 6–8 days. For the experiments, cells were rinsed once and then incubated with either MAO inhibitor pargyline (100 μM, determined from previous experiments) or 1 μM safinamide for 20 min in serum-free media, followed by the addition of H_2_O_2_ (100 μM) or tyramine (100 μM) for 45 min. Working concentration of safinamide was determined in preliminary experiments using a 0.5–4 μM concentration range, which indicated 1 μM as the lowest dose with highest response (data not shown). Finally, myoblasts were loaded with MTR (20 nM) for 15 min. All the steps were carried out at 37°C with 5% CO_2_. Myoblasts were then washed twice, and the chambered coverslips were transferred to a Leica DMI6000B microscope, equipped with a digital camera. Mitochondrial fluorescence was measured in 10–15 random fields per chamber, and data were averaged per field. For each group, four to six chambers were analyzed. Experiments with the various compounds were always performed in parallel with their respective untreated controls. Fluorescence emission was monitored by using 560 ± 20 nm excitation and 645 ± 37 nm emission filter setting. Data were acquired and analyzed using Metafluor software (Universal Imaging).

### Mitochondrial Membrane Potential and Complex I Activity

Mitochondrial membrane potential was measured based upon the accumulation of tetramethylrhodamine methyl ester (TMRM, Molecular Probes) as previously described (Sorato et al., 2014). Myotubes from DMD patients and healthy donors were obtained as described above and treated with H_2_O_2_ (100 μM) for 30 min in the absence or presence of safinamide pre-treatment (1 μM, added 20 min before hydrogen peroxide). Medium was then replaced with serum-free media supplemented with 25 nM TMRM for 30 min, and cellular fluorescence images were acquired with a Leica DMI6000B microscope. Data were acquired and analyzed using Metafluor software (Universal Imaging). For detection of fluorescence, 540 ± 20 nm excitation and 590 nm long-pass emission filter settings were used. Clusters of several mitochondria were identified as regions of interest (ROI), and fields not containing cells were taken as the background. To exclude artifacts due to the different loading capacity of the various cells, which could be erroneously interpreted as ΔΨm differences, sequential digital images were acquired before and after the addition of carbonyl cyanide-*p*-trifluoromethoxy-phenylhydrazone (FCCP, 4 μM), a protonophore that fully depolarizes mitochondria. ΔΨm was estimated as the difference in TMRM fluorescence intensity before and after FCCP of ROI from at least 30 cells. Experiments with the different agents as described above were always performed in comparison with untreated cells. Fluorescence values from the latter were considered as 100%. Complex I activity was measured in myotube lysates using the specifically designed spectrophotometric assay described in Spinazzi et al. (2012).

### Data Analysis and Statistical Procedures

Data are expressed as the mean ± SEM. Analyses were carried out using ANOVA ordinary one-way test followed by Tukey’s multiple comparison test using the Prism software; values with *p* < 0.05 were considered significant.

## Results

### Treatment of mdx Mice With Safinamide Ameliorates Muscle Pathology

**FIGURE 1:**
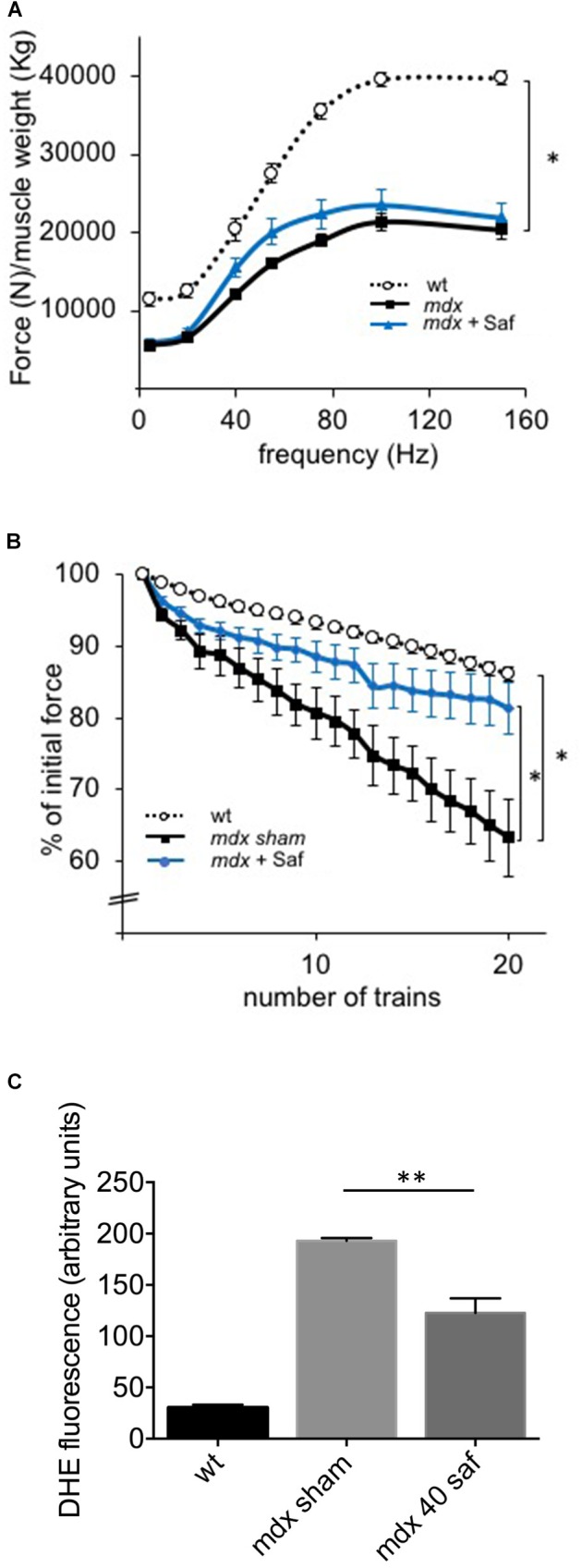
One-week treatment with safinamide rescued functional alterations and oxidative stress in *mdx* mice. Three-month-old *mdx* mice were treated for 7 days with intra-peritoneal injections of either safinamide (40 mg/kg/day) or vehicle alone (*n* = 8 for each condition). Eight syngeneic, wild-type animals were used as reference. **(A)** *In vivo* force–frequency curves of GC muscles from sham- or safinamide-treated *mdx* mice showed the expected drop in normalized force compared to wild type, but no significant improvement with safinamide. **(B)** *In vivo* recordings during eccentric contractions showed a reduced force drop (measured as percent of initial force after 20 eccentric contraction *in vivo*) in safinamide-treated compared to sham-treated animals. **(C)** Quantification of DHE fluorescence in gastrocnemius muscle cryosections from mice treated with vehicle alone (sham) or with safinamide showed reduced ROS accumulation after safinamide treatment. **(C)** Value from wild-type animals was statistically different from those found in both *mdx* counterparts; significance bars were omitted for the sake of chart readability. Data are expressed as mean ± SEM; ^∗^*p* < 0.05 and ^∗∗^*p* < 0.01.

In the first round of *in vivo* experiments, adult *mdx* mice (3-month-old) were subjected to daily intraperitoneal delivery of safinamide (40 mg/kg/day) for 1 week. At the end of the treatment, mice were subjected to a set of *in vivo* force measurements after electrical stimulation of the sciatic nerve as described in the M&M section, after which they were sacrificed to harvest specific muscles for further analyses. Even though safinamide did not lead to significant improvements in normalized force (**Figure 1A**), treated animals showed a strong and significant reduction in force drop upon eccentric contractions, a typical hallmark of dystrophic muscles (**Figure 1B**). Upon harvesting, *gastrocnemius* muscles were then sectioned and subjected to DHE staining to quantify intracellular ROS. Our findings indicated a significant reduction in the extent of oxidative stress between treated and control mice (**Figure 1C**).

**FIGURE 2:**
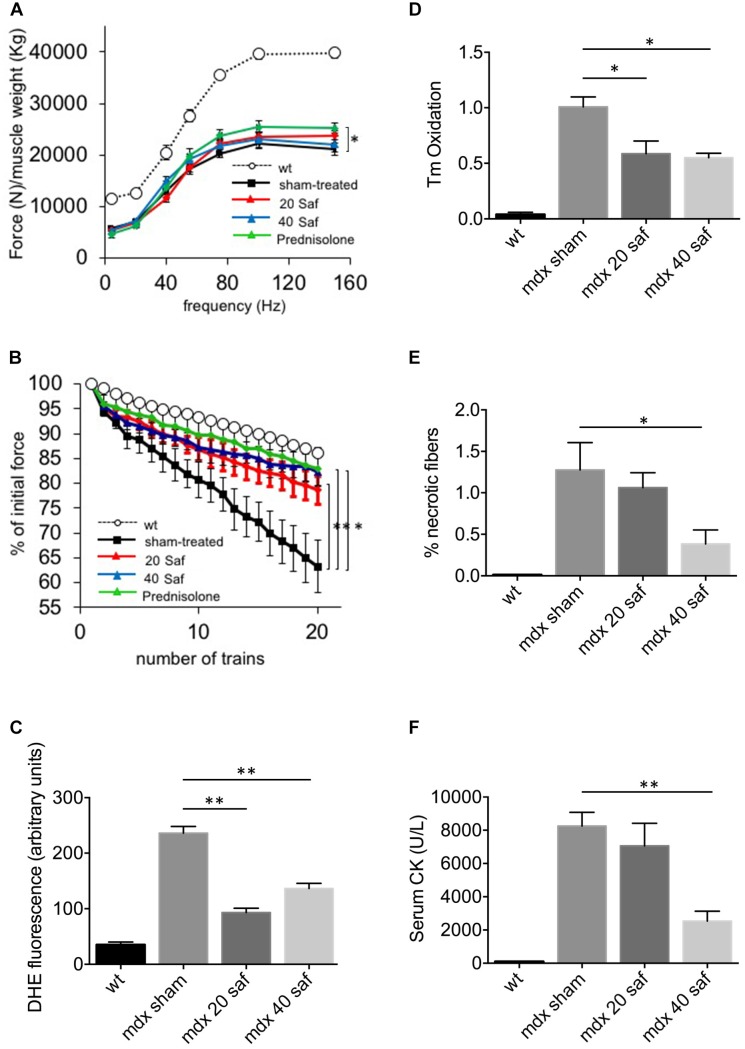
One-month treatment with safinamide rescued functional alterations, oxidative stress, and fiber degeneration in *mdx* mice. Three-month mice were treated for 30 days with intra-peritoneal injections of either safinamide (20 or 40 mg/kg/day), prednisolone, or vehicle alone (*n* = 6 for each condition). Eight syngeneic, wild-type animals were used as reference. **(A)** Force–frequency curves of gastrocnemius muscles from vehicle-treated or safinamide-treated mice showed no significant improvement of normalized force in safinamide-treated animals. **(B)** *In vivo* recordings during eccentric contractions showed a reduced force drop (measured as percent of initial force after 20 eccentric contraction *in vivo*) in safinamide-treated compared to sham-treated animals. **(C)** Quantification of DHE fluorescence in gastrocnemius muscle cryosections from sham- and safinamide-treated mice. **(D)** Oxidation of tropomyosin in gastrocnemius muscles from sham- and safinamide-treated mice. *Y* axis reports the amounts of oxidized tropomyosin normalized to that present in sham-treated animals. **(E)** Quantification of the necrotic fibers by means of immunohistochemical staining for IgG in gastrocnemius muscle from sham- and safinamide-treated mice. *Y* axis reports the amounts of oxidized tropomyosin normalized to that present in sham-treated animals. **(F)** Quantification of the levels of creatine kinase (CK) in the blood of sham- and safinamide-treated mice. **(A,C–F)** values from wild-type animals were always statistically different from those found in all *mdx* counterparts; significance bars were omitted for the sake of chart readability. Data are expressed as mean ± SEM; ^∗^*p* < 0.05 and ^∗∗^*p* < 0.01.

A second cohort of *mdx* mice was then treated for a longer period, 1 month, and with two different doses of safinamide (20 and 40 mg/kg/day), with the same administration protocol. Once again, muscle function was then assessed *in vivo* in terms of resistance to eccentric contractions and of normalized force production. Both safinamide regimens induced a significant improvement in the resistance against eccentric contractions (**Figure 2A**), while no significant improvement in normalized force could be found in safinamide-treated *mdx* mice compared to controls (**Figure 2B**). In parallel, a set of *mdx* mice was treated for the same length of time with the glucocorticoid prednisolone (i.p., 2 mg/kg/day), the standard treatment for DMD patients which is also effective in mice (Keeling et al., 2007 and references therein). Interestingly, the functional improvement found in these mice was comparable to that found with safinamide in the eccentric contraction tests, while the effect on normalized force reached statistical significance only in prednisolone-treated mice (**Figures 2A,B**). DHE staining on *gastrocnemius* sections were in agreement with the results of the short-term experiments, as they confirmed that both safinamide regimens significantly reduced intracellular ROS (**Figure 2C** and **Supplementary Figure S1**). In addition, in these animals, we measured the levels of tropomyosin oxidation, in order to verify whether MAO-B-dependent ROS accumulation could cause oxidative modifications of myofibrillar proteins. The rationale of this choice relied on our previous observation that, among myofibrillar proteins, tropomyosin is particularly susceptible to oxidative stress (Canton et al., 2006; Menazza et al., 2010). We observed a marked reduction in oxidation of tropomyosin with both safinamide treatments (**Figure 2D** and **Supplementary Figure S1**). To investigate whether other pathological features found in dystrophic muscles had been affected by the 1-month treatment, we then quantified the number of necrotic fibers present in the gastrocnemius muscles of treated and control mice by immunofluorescence, using an anti-murine IgG antibody to label fibers containing serum immunoglobulin in their sarcoplasm. In this case, safinamide at 40 mg/kg led to a significant reduction in the percentage of necrotic fibers (**Figure 2E** and **Supplementary Figure S2**). Since necrosis of muscle fibers leads to a steep increase in circulating CK, making it a hallmark of Duchenne dystrophy in humans and in mice, we also compared serum CK levels between treated and untreated animals. **Figure 2F** shows how the treatment with 40 mg/kg safinamide led to an almost fourfold reduction of CK activity in serum. Finally, we also performed standard histological evaluations of the sections (by means of H&E staining), analyzing the percentage of centrally nucleated fibers and the distribution of fiber diameters, both indicators of the regenerative status of a striated muscle. No differences were found between treated and control animals in either case (**Supplementary Figure S2**).

### Safinamide Reduces Oxidative Stress and Mitochondrial Dysfunction in Murine Dystrophic Myoblasts

**FIGURE 3:**
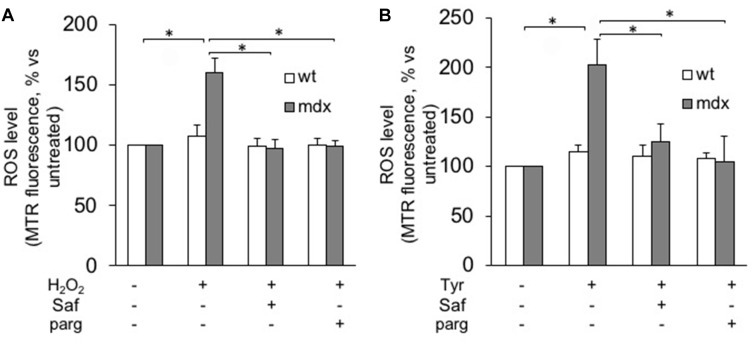
MAO-B inhibition reduces ROS accumulation in response to oxidative stress in myoblasts from *mdx* mice. Myoblasts from *mdx* and *wild-type* (wt) mice were incubated for 1 h with **(A)** H2O2 (100 μM) or **(B)** tyramine (Tyr, 100 μM) in the absence or in the presence of 1 μM safinamide or 100 μM pargyline (parg). ROS levels were assessed by Mitotracker Red CM-H_2_XRos (MTR, 25 nM). Data expressed as the MTR fluorescence after 1 h from the addition of H2O2 (or Tyr) were normalized to the values obtained in the absence of stimuli for each sample. Data are the mean ± SEM of three experiments, each using cells originating from different preps. ^∗^*p* < 0.05 and ^∗∗^*p* < 0.01.

Next, we isolated myoblasts from *mdx* and *wild-type* mice and proceeded to evaluate their susceptibility to oxidative stress. To this aim, we used a protocol that we and others had already exploited to mimic the oxidative stress occurring in several disease states (Disatnik et al., 1998; Sorato et al., 2014), in which proliferating myoblasts are exposed to hydrogen peroxide (100 μM) for 1 h in the presence or in the absence of MAO inhibitors. The rationale of such treatment is that both muscles from *mdx* mice and DMD patients are constantly exposed to high levels of ROS produced by the infiltrated phagocytic cells (Chen and Nunez, 2010). Among them, hydrogen peroxide is more stable and diffuses through membranes. In our experiments, we tested safinamide at a 1-μM concentration, as well as pargyline, a strong, first-generation inhibitor of both MAO-A and MAO-B, at 100 μM. After the challenge with hydrogen peroxide, ROS levels were assessed by exposing muscle cells to a redox sensitive fluorescent probe (Mitotracker, CM-H_2_XRos) and measuring the corresponding fluorescence emission (Sorato et al., 2014). Our data show that this protocol greatly increased ROS levels in *mdx* but not in *wild-type* myoblasts and, importantly, such accumulation was suppressed by treatment with safinamide and pargyline (**Figure 3A** and **Supplementary Figure S3**). The effect elicited by MAO-B inhibition appears superimposable to that induced by inhibiting both MAO isoforms. It should be noticed that no obvious morphological signs of cell death were detected in any condition with either *mdx* or *wild-type* cells (data not shown), a somewhat expected observation given the combination of relatively low hydrogen peroxide concentration and short time of exposure.

Next, we assessed the role of MAO and its activity in an alternative way, by incubating our murine myoblasts with tyramine, a typical MAO substrate whose oxidative deamination generates hydrogen peroxide as a by-product. Once again, higher levels of ROS were detected in *mdx* myoblasts as compared with *wild-type* cells, and they were blunted by safinamide treatment (**Figure 3B**). Finally, it should be noticed that in the absence of the above-described stressors no significant differences in ROS levels were observed between *mdx* and *wild-type* myoblasts (not shown).

### Safinamide Reduces Oxidative Stress and Mitochondrial Dysfunction in Human Dmd Myoblasts and Myotubes

The efficacy of safinamide in reducing ROS formation was then tested in myoblasts and myotubes obtained from cultured human myoblasts. Specifically, we used primary cultures derived from muscle biopsies (one healthy donor and two DMD patients), as well as immortalized myoblasts (Mamchaoui et al., 2011) (two healthy donors and two DMD patients, see **Table 1** for details). The rationale of using both primary and immortalized cells derived from the fact that we wished to rule out the possible effect of two potentially relevant drawbacks commonly found in the former, namely a relatively low myogenicity and the unavoidable presence in the cultures of varying amounts of non-myogenic cells (Mamchaoui et al., 2011).

**Table 1:** Patients’ description.

	Age at time of biopsy	Type of mutation
DMD 1 (primary cells)	24 months	Del ex 44
DMD 2 (primary cells)	24 months	Del ex 8–17
DMD 3 (immortalized cells)	20 months	Del ex 48–50
DMD 4 (immortalized cells)	20 months	Del ex 45–52


**FIGURE 4:**
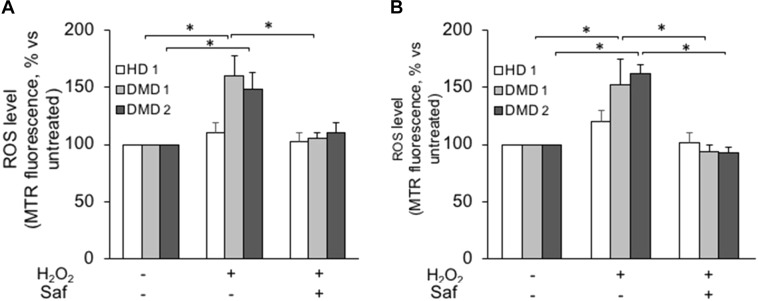
MAO-B inhibition decreases ROS accumulation in response to oxidative stress in primary cultured myoblasts and myotubes from DMD patients. Primary myoblasts **(A)** or myotubes **(B)** from one healthy donor (HD 1) and DMD patients (DMD 1 and DMD 2) were loaded with Mitotracker Red CM-H_2_XRos (MTR, 25 nM). Oxidative stress was induced by H_2_O_2_ addition (100 μM) with or without 1 μM safinamide, as a 20-min pre-treatment. Data expressed as the MTR fluorescence after 1 h from H_2_O_2_ were normalized to the values obtained in the absence of stimuli for each sample. Data are the mean ± SEM of three experiments per cell prep. ^∗^*p* < 0.05.

Similarly to what had been done with murine cells, human myogenic cultures were challenged with hydrogen peroxide in the presence or in the absence of MAO inhibitors. In this case, though, the experiments were performed not only on cultured myoblasts but also on differentiated myotubes. For the former, **Figure 4A** shows how ROS levels increased upon exposure to hydrogen peroxide in primary DMD myoblasts and, importantly, that once again they were brought back to the levels found in non-dystrophic cells by the treatment with safinamide. Importantly, the hydrogen peroxide challenge did not affect myoblasts from healthy donors, in accordance with our present data in murine cells and with our previous data in human cells (Sorato et al., 2014). The same type of experiment was then performed on myotubes differentiated from the same cell preparations. Similarly to myoblasts, upon exposure to hydrogen peroxide DMD myotubes were found to be more susceptible to oxidative stress than myotubes from healthy donors and the rise in ROS levels was prevented by safinamide (**Figure 4B**).

**FIGURE 5:**
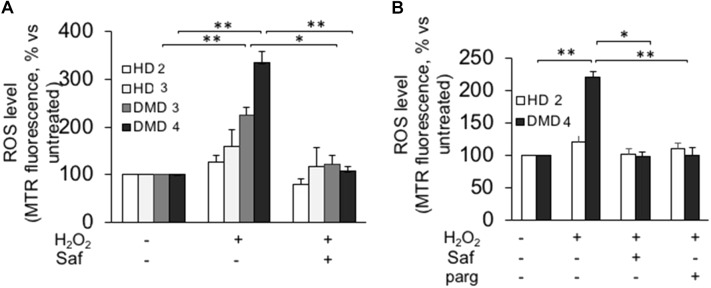
MAO-B inhibition reduces ROS accumulation in response to H_2_O_2_ in immortalized cell lines from DMD patients. Immortalized myoblasts **(A)** and myotubes **(B)** obtained from healthy donors (HD 2 and HD 3) and DMD patients (DMD 3 and DMD 4) were loaded with Mitotracker Red CM-H_2_XRos as described in **Figure 4**. Oxidative stress was induced by H_2_O_2_ addition (100 μM) in the absence or presence of 1 μM safinamide or 100 μM pargyline, as a 20 min pre-treatment. Data expressed as the MTR fluorescence after 1 h from H_2_O_2_ were normalized to the values obtained in the absence of stimuli for each sample. Data are the mean ± SEM of three experiments per cell prep. ^∗^*p* < 0.05 and ^∗∗^*p* < 0.01.

Next, we investigated whether these findings held true in cultures comprising only myoblasts, thanks to the use of the above-mentioned immortalized cell lines. Upon hydrogen peroxide stimulation, both myoblasts (**Figure 5A** and **Supplementary Figure S4**) and myotubes (**Figure 5B** and **Supplementary Figure S4**) from immortalized DMD cells displayed increased ROS levels, which were reduced to levels comparable to those found in controls by the inhibition of MAO-B. As in primary cultures, hydrogen peroxide challenge had no effect on the ROS levels found in cells derived from healthy donors. These finding not only confirmed the increased susceptibility of DMD cells to oxidative stress but also indicated that the biological mechanism(s) leading to such phenomenon were indeed intrinsic to the myogenic cells and did not depend on the interaction with the non-myogenic fraction of primary cultures.

**FIGURE 6:**
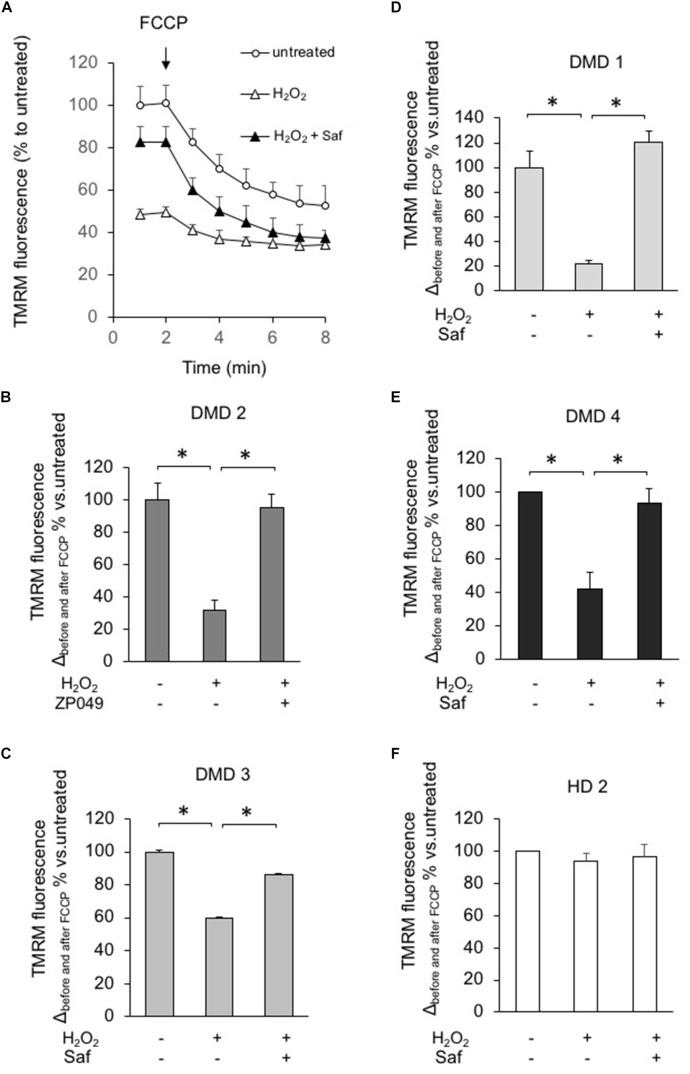
MAO-B inhibition reduces mitochondrial dysfunction in response to H_2_O_2_ in myotubes from DMD patients. DMD or HD myotubes (from primary and immortalized cells) were exposed to H_2_O_2_ (100 μM) in the absence or presence of 1 μM safinamide, as a 20 min pre-treatment and then loaded with TMRM (25 nM) to monitor the mitochondrial membrane potential ΔΨm. **(A)** Representative kinetics of TMRM fluorescence intensity in one experiment with primary myotubes from patient DMD2. Single data points are the average of at least 15 individual myotubes. When indicated, FCCP (4 μM) was added to collapse ΔΨm. In the absence of safinamide, H_2_O_2_ treatment caused a drastic drop in the initial membrane potential, which led to a small difference in ΔΨm before and after FCCP. **(B–F)** Charts representing the variation in TMRM fluorescence intensities obtained in each condition before and after FCCP; one chart per cell type. Values in *Y* axis are expressed as percentage, considering the value in untreated cells as 100%. Each cell type was analyzed in two independent experiments. ^∗^*p* < 0.05.

Finally, we also assessed the effect of safinamide on mitochondrial membrane potential, by means of the fluorescent probe TMRM, after inducing oxidative stress with hydrogen peroxide. Similarly to what was seen with ROS levels, the presence of hydrogen peroxide had a marked effect in DMD myotubes – namely, a reduction in mitochondrial membrane potential – but not in control cells. Once again, treatment with safinamide prevented such drop (**Figure 6**). We hypothesized that the increased levels of ROS in DMD myotubes could affect mitochondrial membrane potential by decreasing the activity of the respiratory chain. For this reason, we evaluated the activity of Complex I in myotubes from a healthy donor and a DMD patient used for membrane potential determination, but found no differences between the two (data not shown).

## Discussion

Many biological and medical issues related to muscular dystrophies are far from being conclusively addressed, thus hampering the development of adequate interventions. The identification and exploitation of novel pharmacological agents is, therefore, urgently needed to improve clinical management of Duchenne patients. The data, we present here, investigate the mitochondrial enzyme MAO-B as a promising target toward such goal. In particular, building on our previous findings, the present study provides two novel aspects: (i) in the context of DMD, MAO-B plays a key role in determining oxidative stress, and (ii) a novel inhibitor for this isoform significantly improves functional impairment occurring in *mdx* mice and can protect myotubes of DMD patients from mitochondrial dysfunction.

The first part of our study has been carried out *in vivo*, analyzing both force production and histo-pathological features. For the former, we investigated two parameters: force loss upon eccentric contractions and normalized force. The rational of this choice is that one of the main aspects of DMD, as well as other muscular dystrophies, is the susceptibility of skeletal muscles to activity-induced muscle damage (Blaauw et al., 2010; Rader et al., 2016). While the exact molecular mechanism(s) behind the increased sensitivity to eccentric contractions is not known, multiple reports have shown that reducing oxidative stress can be beneficial (Whitehead et al., 2008). Furthermore, a central role of mitochondria in activity-dependent increases in pathological oxidative stress has also been reported in a different kind of myopathy, i.e., central core disease (Durham et al., 2008). Our data indicate that in *mdx* mice the MAO-B-dependent increase in mitochondrial ROS production plays an important role in decreasing force production during repeated eccentric contractions. Interestingly, the positive effect of inhibiting MAO-B could be seen even after a short (1 week) treatment, suggesting a mechanism that does not involve extensive tissue remodeling. While specific experiments will be needed to clarify this point, it is tempting to hypothesize that the molecular mechanism(s) involved in the positive effect of safinamide on rescuing activity-induced force loss are related to an increase in calcium levels within the mitochondria due to the opening of stretch-dependent calcium channels (Yeung et al., 2005). As for the normalized force production, treated animals consistently yield higher values than controls, but the differences never reached statistical significance, hence suggesting that ROS levels are not a main player in the mechanism(s) leading to the decreased isometric force in *mdx* muscles, or longer treatment periods are required to induce significant changes.

Analysis of muscle sections in treated animals confirmed that safinamide did decrease ROS levels in the fibers of treated animals as well as the oxidative status of a key component of the contractile apparatus (tropomyosin), hence providing new insights on the molecular mechanism(s) linking dystrophin deficiency to protein oxidation. Under the histopathological point of view, our data also showed that MAO-B inhibition provided a significant protection from fiber degeneration, as demonstrated by the decrease in CK serum levels and by the lower count of necrotic fibers. These findings suggest that the end mechanism(s) leading to fiber death are linked, at least in part, to the excessive production of ROS. On the other hand, our analyses of fiber size and percentage of central nuclei did not show any effect of safinamide, thereby suggesting that, at least in our experimental setting, changes in the levels of intracellular ROS do not affect the processes of muscle regeneration. In order to confirm such hypothesis, though, specific experiments with animals treated with safinamide at a younger age (i.e., before or during the occurrence of the initial massive degeneration/regeneration bout) will be needed.

In view of a possible drug repurposing, it is also important to stress how the functional benefits obtained with safinamide were quite similar to those seen in our animals upon treatment with prednisolone, one of the glucocorticoids that at present represent the only standard therapy for Duchenne patients. Such finding is even more relevant in view of the fact that while glucocorticoids are usually effective in delaying loss of ambulation (McDonald et al., 2018), their chronic use present severe side-effects (e.g., severe bone demineralization) that in many cases can actually force discontinuation. On the other hand, the new generation of MAO-B inhibitors to which safinamide belongs exerts far fewer unwanted side-effects, not only compared to glucocorticoids but also to the older, non-specific MAO inhibitors. MAO-B molecular structure has been identified at high resolution (Binda et al., 2002, 2004), thus allowing the design of highly specific inhibitors that have the advantage to avoid the risk of hypertensive crises that is associated to inhibition of the MAO-A isoform. Indeed, MAO-B specific inhibitors are currently used in treatment of several neurological disorders (Youdim et al., 2006).

The involvement of mitochondria in DMD pathogenesis has been reported both in patients and in animal models, for instance in terms of deficit of electron transport respiratory chain components and of enzymes of the tricarboxylic acid cycle (Timpani et al., 2015). As a downstream consequence, the ATP levels in these muscles were found to be severely reduced causing altered ionic homeostasis and oxidative stress (Rodriguez and Tarnopolsky, 2003; Ramadasan-Nair et al., 2014). Our *in vitro* experiments with murine and human myoblast cultures clearly indicated that cells derived from dystrophic muscles have a higher susceptibility to oxidative stress compared to non-dystrophic counterparts. To the best of our knowledge, this is the first study that explores the source of excess ROS in myogenic cultures isolated from DMD patients, establishing a causal relationship between MAO-B-dependent increased ROS levels and mitochondrial dysfunction.

A remarkable aspect of our findings is that in dystrophic cells higher sensitivity to oxidative stress appears to be independent from dystrophin expression, given that in *in vitro* cultures the dystrophin gene is expressed in myotubes but not in myoblasts. This finding is in agreement with previous studies showing that the impaired metabolism observed in *mdx* myoblasts was independent of dystrophin-deficiency (Onopiuk et al., 2009). In this regard, it should be noticed that recent evidences have demonstrated the expression of dystrophin in activated satellite cells, where it plays an important role in establishing their capability for asymmetric division (Dumont et al., 2015). The authors also confirmed the absence of dystrophin in proliferating myoblasts and while they did not investigate the exact timing and mechanism of disappearance, they showed that already at 72 h after activation of satellite cells dystrophin could be seen only in a small proportion of them. For these reasons, our evidences indicate that myoblasts derived from a dystrophic muscle maintain a series of metabolic alterations even after prolonged *in vitro* cultures; the presence of specific epigenetic markings originated *in vivo* in the context of dystrophic muscle seems to be a likely explanation, although specific experiments will be needed to confirm this hypothesis. In this regard, it is interesting to notice that Disatnik et al. (1998) reported no increased sensitivity to oxidative stress in myoblasts cultures prepared from newborn *mdx* mice, a developmental stage in which there is no overt dystrophic phenotype. The use of both primary and immortalized cells allowed us to demonstrate that the molecular mechanisms leading to the peak of oxidative stress present in DMD cells take place specifically within myoblasts. By the same token, the fact that in our assays immortalized cells showed the same phenotype found in primary cultures further confirms that the former are a reliable alternative to the latter, while presenting clear advantages in terms of long-term availability and reproducibility.

At present, our findings do not provide indications about the mechanisms linking MAO-mediated oxidative stress to mitochondrial depolarization. As a preliminary attempt, we verified whether the accumulation of ROS in DMD myotubes affected the activity of NADH dehydrogenase, the complex of the respiratory chain that is more susceptible to oxidative stress, but found no differences between healthy donor and DMD cells. Further studies will be aimed at characterizing the molecular targets of MAOB-dependent ROS increased levels.

Overall, these results provide clear evidence of safinamide efficacy in reducing oxidative stress in dystrophic skeletal muscle. Importantly, though, safinamide treatment should not be considered just another case of antioxidant therapy, as it does not act as a general ROS scavenger. Such an approach could actually be cause of some concerns, considering that small amounts of ROS are required for intracellular signaling. On the contrary, safinamide act by specifically preventing the formation of a subset of mitochondrial ROS that becomes disproportionate in pathological conditions because of MAO over activation.

## Author Contributions

BB and MC contributed conception and design of the study. LV, MM, ES, LN, GF, MA, BB, and MC performed the experiments and the subsequent data analyses. VM provided the immortalized cells and contributed to the drafting of the manuscript. MA and LS contributed conception of specific experiments. LV, MC, and BB wrote the manuscript.

## Conflict of Interest Statement

MC was co-holder (with three other colleagues who did not participate to the present study) of a patent covering the use of a specific class of MAO-B inhibitors for the therapy of muscular dystrophies. As of April 2018, such patent, which does not cover the molecule presented in the present study, has been sold by the University of Padova to a private company. The remaining authors declare that the research was conducted in the absence of any commercial or financial relationships that could be construed as a potential conflict of interest. The reviewer MM and handling Editor declared their shared affiliation at the time of the review.

## Footnotes

1 https://imagej.net/ImageJ1
