# Supplementary figures and images for: Drug Repurposing for Duchenne Muscular Dystrophy: The Monoamine Oxidase B Inhibitor Safinamide Ameliorates the Pathological Phenotype in mdx Mice and in Myogenic Cultures From DMD Patients
Source: Front Physiol. 2018 Aug 14;9:1087. doi: 10.3389/fphys.2018.01087 (PMC6102489; doi:10.3389/fphys.2018.01087)

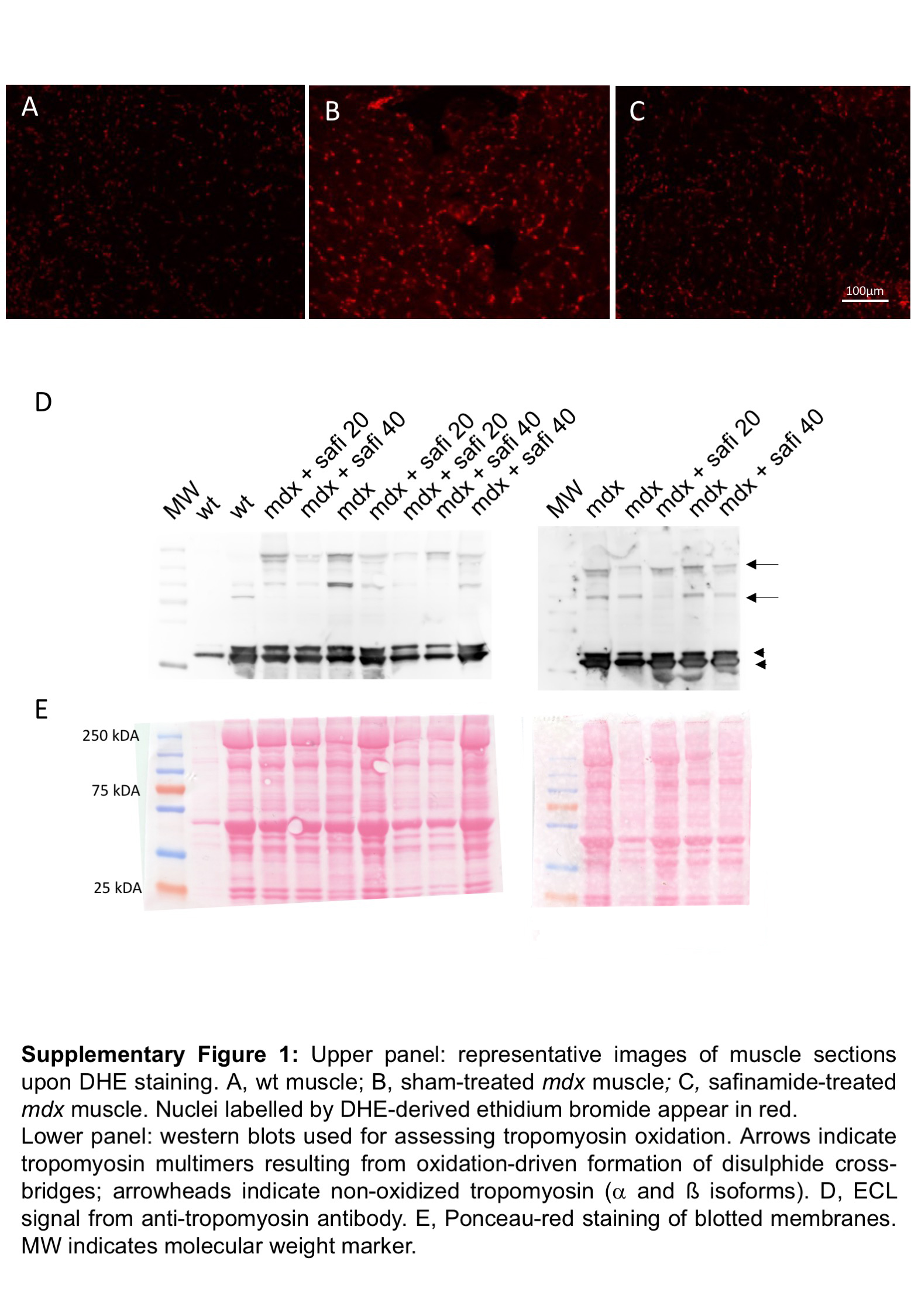

Supplement: Supplementary file 1 [file Image_1.JPEG]

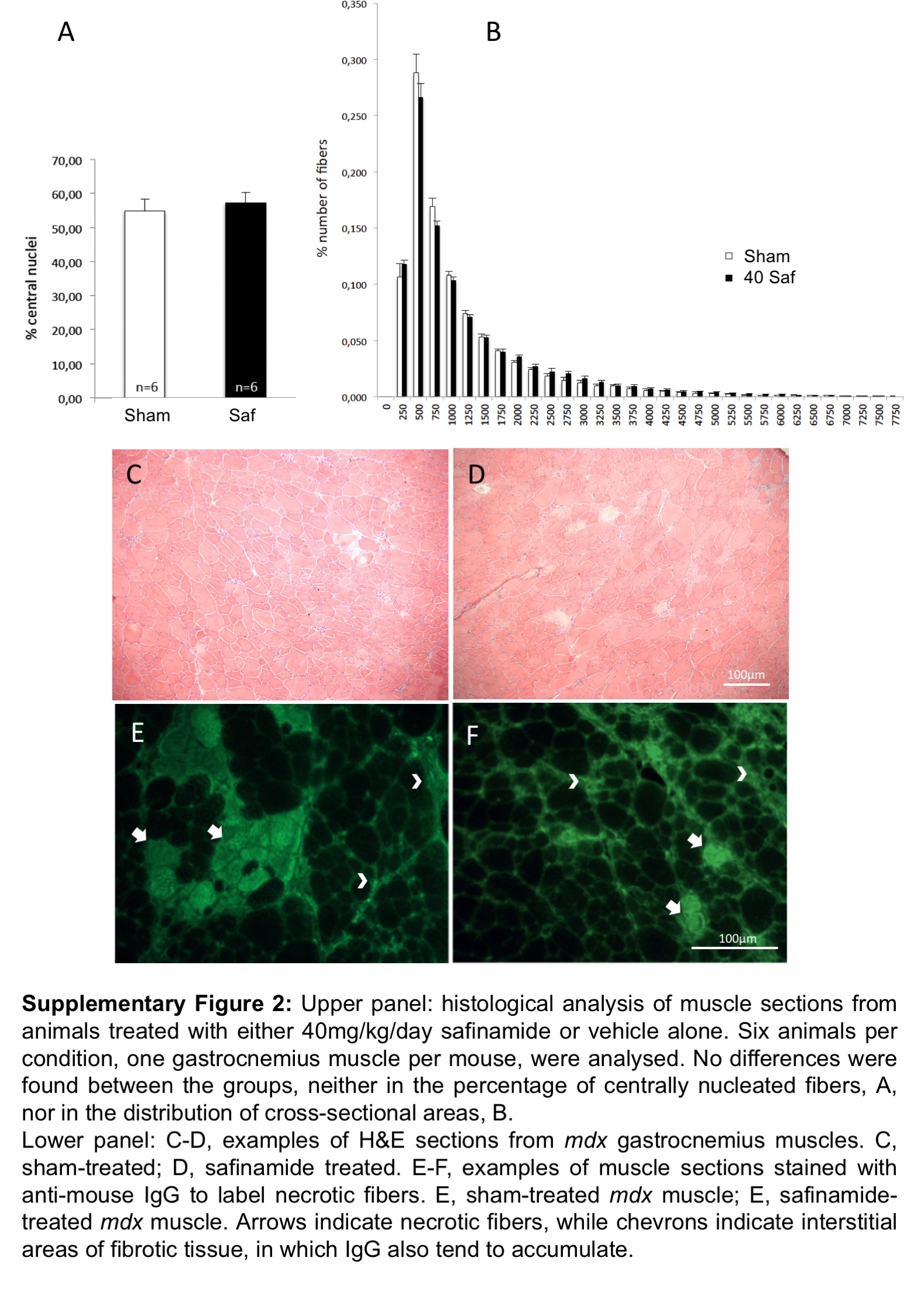

Supplement: Supplementary file 2 [file Image_2.JPEG]

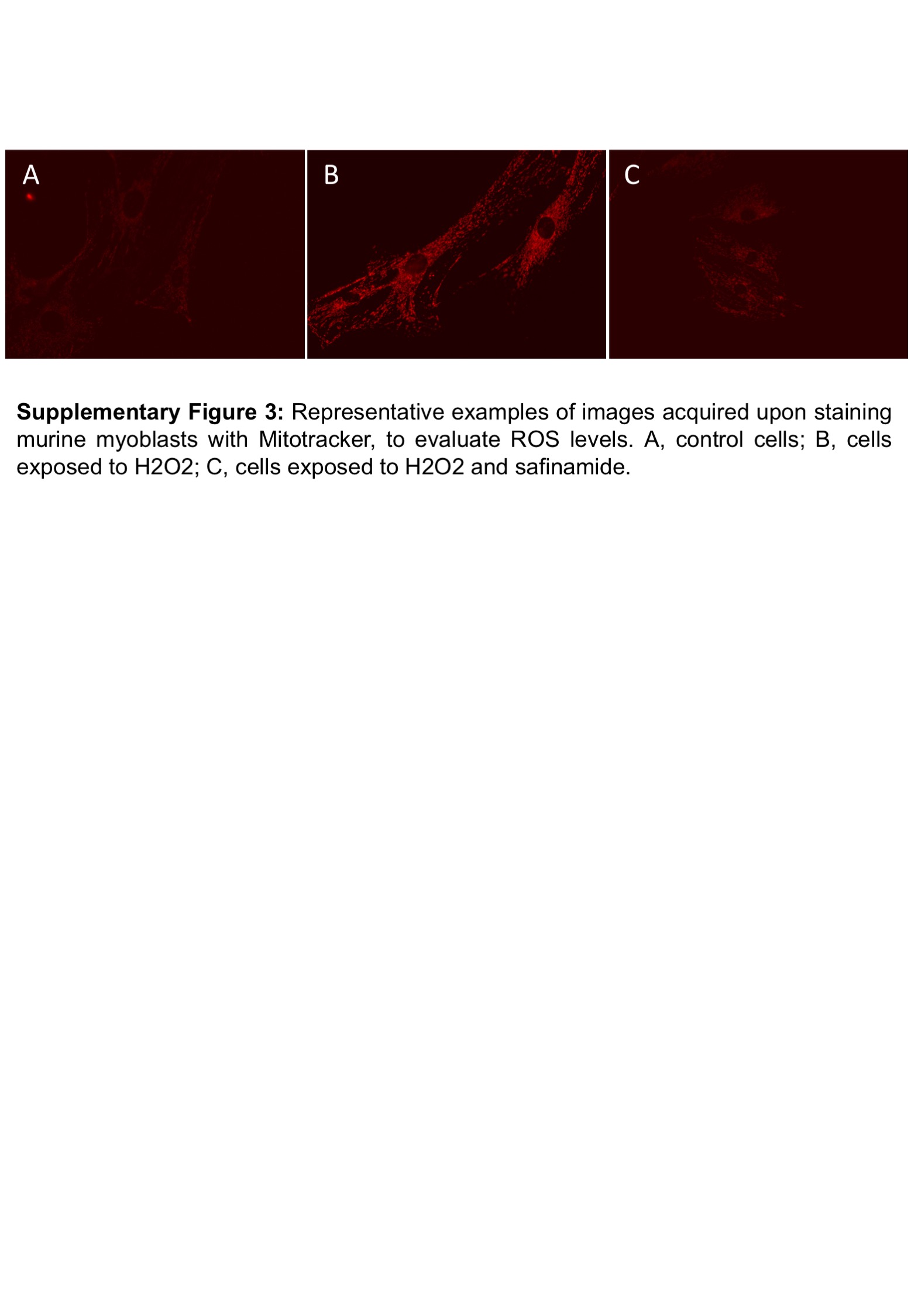

Supplement: Supplementary file 3 [file Image_3.JPEG]

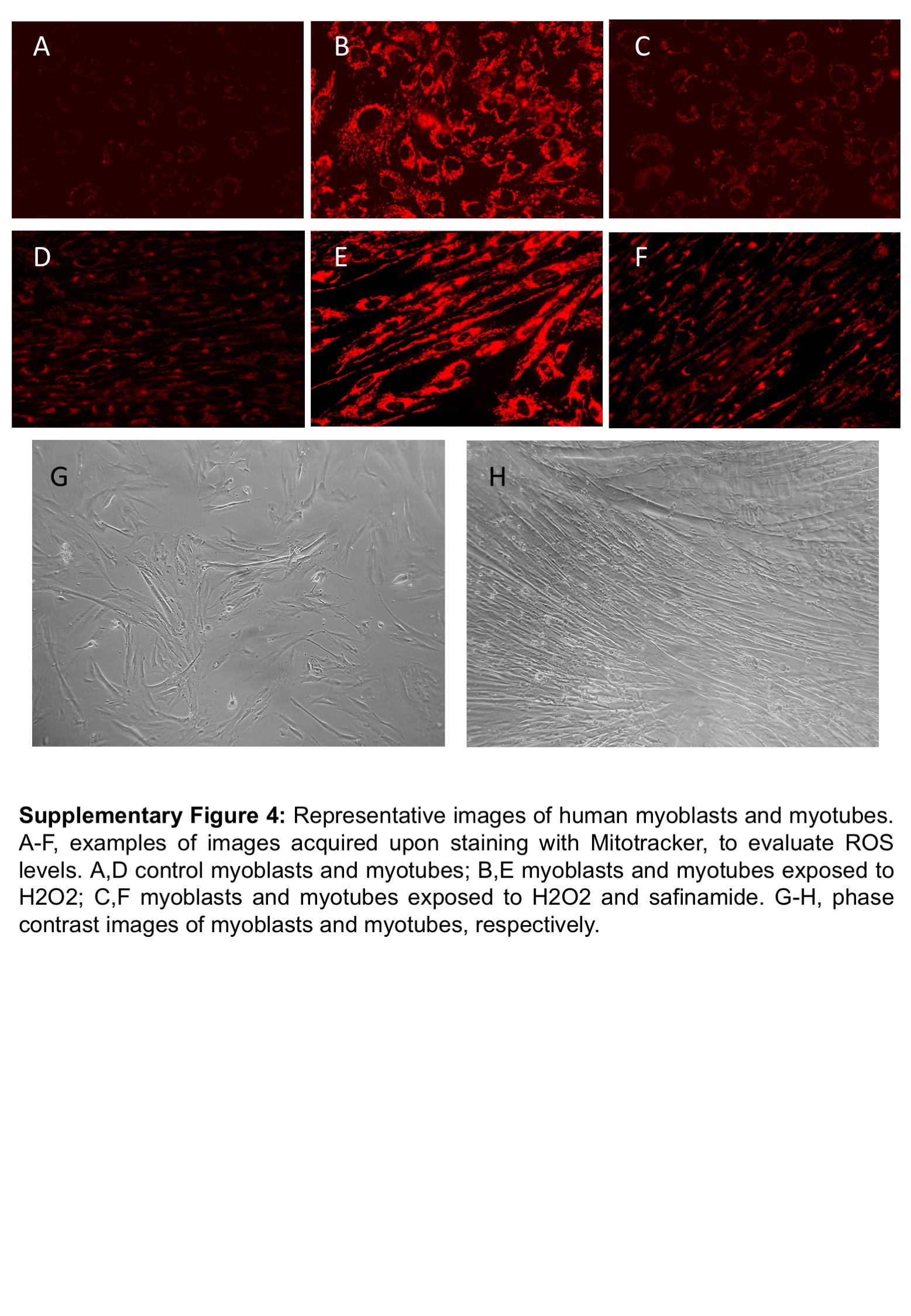

Supplement: Supplementary file 4 [file Image_4.JPEG]
